# Unlocking the therapeutic potential of ATR inhibitors: Advances, challenges, and opportunities in cancer therapy

**DOI:** 10.1002/ctm2.70397

**Published:** 2025-07-17

**Authors:** Tejaswini P Reddy, Timothy A. Yap

**Affiliations:** ^1^ Department of Internal Medicine Baylor College of Medicine Houston Texas USA; ^2^ Department of Investigational Cancer Therapeutics (Phase I Program) The University of Texas MD Anderson Cancer Center Houston Texas USA; ^3^ Therapeutics Discovery Division The University of Texas MD Anderson Cancer Center Houston Texas USA

## 1 INTRODUCTION

**FIGURE 1:**
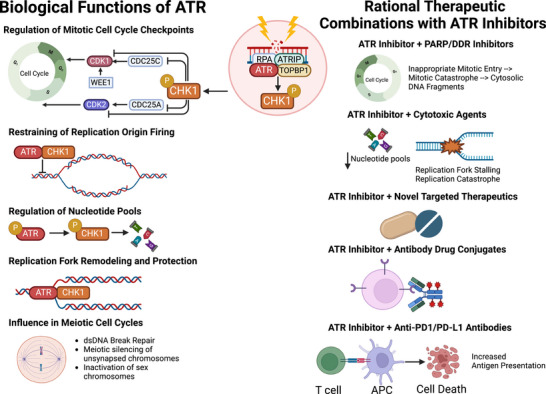
Schematic of biological functions and ATR and rational combination therapeutic strategies with ATR inhibitors. In response to replication stress, ATR‐Chk1 signalling is involved in multiple biological functions including regulation of mitotic checkpoints via degradation of M‐phase inducer phosphatase (CDC25A/C), which negatively regulates cyclin‐dependent kinases 1 and 2 (CDK1/2), leading to activation of G2/M and intra‐S checkpoints. The ATR/Chk1 proteins also restrains firing of replication forks, increases synthesis and reduces degradation of nucleotide pools, protects exposed replication forks from nucleases and regulates meiotic cell cycle. Taking these biological functions into consideration, there are multiple rational combination strategies with ATR inhibitors, including ones with antibody–drug conjugates, anti‐PD‐1 antibodies or anti‐PD‐L1 antibodies, other inhibitors of the DNA damage response (DDR; including poly(ADP‐ribose) polymerase, PARP), cytotoxic agents and novel targeted therapies, such as phosphatidylinositol 3‐kinase inhibitors.

The DNA damage response (DDR) and replication stress (RS) response networks consist of a highly integrated group of proteins crucial for maintaining genomic integrity and cellular survival. These networks manage DNA replication, repair, cell cycle transitions and apoptosis.^1^ Primary regulators of the DDR are phosphoinositide 3‐kinase related protein kinases (PIKKs), notably ataxia telangiectasia mutated (ATM) and Rad‐3 related (ATR). ATR can be activated in response to extensive single‐stranded DNA breaks (ssDNA) at stalled replication forks and other forms of replication stress, triggering downstream reactions, such as the phosphorylation of serine‐threonine kinase Chk1. The ATR‐Chk1 signalling cascade plays a key role in various biological processes, including mitotic cell cycle checkpoint regulation, replication fork stabilization and remodelling, suppression of replication origin firing, regulation of nucleotide pools, meiotic cell cycle progression, and management of cellular mechanical stress and inflammatory processes.^2^ Deficiencies in the DDR and RS response lead to genomic instability, promoting cancer initiation and progression through mutation accumulation. However, these deficiencies also create therapeutic vulnerabilities in cancer cells, allowing for the development of rational molecularly targeted agents against the DDR, such as ATR inhibitors (Figure 1).^3^ This approach has been clinically validated with poly(ADP‐ribose) polymerase (PARP) inhibitors, which have obtained regulatory approval in different tumour types with *BRCA1* or *BRCA2* loss‐of‐function (LOF) mutations.^4^

## 2 TARGETING ATR AND INHIBITORS IN CLINICAL DEVELOPMENT

The rationale for targeting ATR as a therapeutic strategy for various cancer types with DDR defects lies in the fact that ATR inhibition disrupts mechanisms described above that maintain genomic integrity. This disruption leads to genomic instability, causing premature entry into mitosis regardless of RS or DNA damage. This triggers mitotic catastrophe and cellular apoptosis, processes that may be synthetically lethal in cancer cells with DDR defects, such as ATM LOF mutations.^5^ The development of ATR inhibitors originated from studies showing that inhibitory mutations of the ATR kinase domain were primarily hypomorphic or partially inhibitory. ATR also plays other biological roles independent of its kinase activity, such as suppressing mechanical stress and inflammation, which we can therapeutically exploit when combining ATR inhibitors with immunotherapies. Therefore, incomplete or hypomorphic ATR inhibition may represent a promising anti‐cancer therapeutic strategy.^6^, ^7^

ATR inhibitors that have been assessed in clinical trials include the intravenously administered drug berzosertib, and orally administered drugs camosertib, ceralasertib, elimusertib, tuvusertib, ART0380, ATRN‐119, ATG‐018 and IMP9064.^2^ These inhibitors have undergone evaluation in preclinical and clinical settings as monotherapies, as well as in some cases, combination therapies with different agents, including PARP inhibitors, cytotoxic chemotherapies, and immune checkpoint inhibitors.^2^ ATR inhibitors are associated with dose‐dependent myelosuppression, particularly anaemia. ATR inhibitor‐associated anaemia may be linked to the high iron requirements of early‐stage erythroblasts, rendering them sensitive to iron‐dependent reactive oxygen species and ferroptosis.^8^ Efforts to manage ATR inhibitor‐related anaemia include optimizing intermittent dosing schedules (e.g. 3 days on/4 days off) to maintain target engagement while promoting erythroid precursor development and maturation. Overlapping toxicities are especially important considerations with combination therapies that share haematological toxicities (i.e. ATR + PARP inhibitors).

In early‐phase clinical trials, elimusertib and camonsertib monotherapy showed preliminary anti‐tumour activity in patients with advanced solid tumours with homologous recombination repair (HRR) defects.^7^, ^9^ These trials highlighted the importance of patient selection through the use of molecular biomarkers of response when considering treatment with ATR inhibitors.

In a phase I clinical trial of camonsertib in patients with advanced solid tumours with ATR inhibitor sensitizing gene alterations, the overall response rate (ORR) and clinical benefit rate (CBR) across multiple tumour types at a dose of at least 100 mg per day were 13% and 43%, respectively.^7^ Chemogenomic CRISPR‐based datasets were used to identify synthetically lethal ATR inhibitor‐sensitizing HRR alterations (LOF mutations in *ATM, ATRIP, BRCA1, BRCA2, CDK12, CHTF8, FZR1, MRE11, NBN, PALB2, RAD17, RAD50, RAD51B/C/D, REV3L, RNASEH2A, RNASEH2B* or *SETD2*) as a rational basis for patient selection. Compared to other tumour types, patients with ovarian cancer had the highest response rate (25%), highest clinical benefit rate (75%), and longest median progression‐free survival (35 weeks) with camonsertib monotherapy. Notably, these patients were heavily pre‐treated (median six prior lines of therapy), with the majority platinum and/or PARP inhibitor refractory/resistant. One responder had a *BRCA1* reversion alteration (p.E143*  >  p.E143D). Responders also had germline *BRCA1* (*n* = 2) and *RAD51* (*n* = 2) mutations and a somatic *SETD2* mutation (*n* = 1). These findings suggested that ovarian cancers may be specifically vulnerable to ATR inhibitors due to their intrinsically high RS, high frequency of biallelic HRR gene loss, and loss of tumour suppressors. Furthermore, these findings suggested that in a cohort of patients with platinum/PARP‐resistant ovarian cancers harbouring *BRCA1* reversion mutations, the reversion mutation may not completely restore functional HRR processes, rendering these cancer cells still sensitive to ATR inhibitors.

This study also highlighted the complexity associated with evaluating *ATM* LOF in tumours and the concordance between *ATM* allelic status and ATM protein loss found via immunohistochemistry (IHC), as well as the importance of excluding *ATM* mutations derived from clonal haematopoiesis in liquid biopsies.^10^ Preclinical and clinical studies have shown that ATR inhibition is synthetically lethal with LOF of ATM kinase. Despite the detection of biallelic *ATM* LOF being strongly predictive of ATM protein loss, there was a case of a responder with castration‐resistant prostate cancer and a pathogenic biallelic *ATM* R3008H mutation with retained tumoral ATM protein expression. These complexities in determining true *ATM* loss in tumours may be due to the difficulty in defining the pathogenicity of *ATM* LOF mutations, given the large size of the gene and the lack of standardized protocols, antibodies, and expression cut‐offs to define *ATM* loss. These findings underscore the importance of determining the allelic status and pathogenicity of mutations, and their concordance with protein expression to optimize patient selection for ATR inhibitor treatment. In the future, it is possible that a ‘triple approach’ to exploit RS may optimize clinical benefit, for example by targeting ATM‐deficient tumours that have inherently high RS with the combination of a chemotherapy such as irinotecan to induce further RS, in combination with an ATR inhibitor to block cellular rescue from RS.

## 3 CONCLUSIONS

ATR inhibitors show promise as therapeutic agents for cancers with DDR and/or RS response defects. The success of ATR inhibitors depends on appropriate molecular patient selection, highlighting the importance of robust predictive biomarkers and detailed genomic and proteomic profiling to predict response and evaluate mechanisms of resistance. Addressing the complexities of evaluating *ATM* LOF mutations and managing ATR inhibitor‐associated toxicities, such as anaemia, are crucial for optimizing treatment regimens. Future studies should focus on refining patient selection criteria, validating predictive biomarkers of response, and developing strategies to mitigate toxicities, thereby enhancing the clinical utility of ATR inhibitors as monotherapy and in rational combination strategies for treating a range of cancers.
